# CT-Based Radiomic Signature as a Prognostic Factor in Stage IV *ALK*-Positive Non-small-cell Lung Cancer Treated With TKI Crizotinib: A Proof-of-Concept Study

**DOI:** 10.3389/fonc.2020.00057

**Published:** 2020-02-18

**Authors:** Hailin Li, Rui Zhang, Siwen Wang, Mengjie Fang, Yongbei Zhu, Zhenhua Hu, Di Dong, Jingyun Shi, Jie Tian

**Affiliations:** ^1^School of Automation, Harbin University of Science and Technology, Harbin, China; ^2^CAS Key Laboratory of Molecular Imaging, Institute of Automation, Chinese Academy of Sciences, Beijing, China; ^3^School of Artificial Intelligence, University of Chinese Academy of Sciences, Beijing, China; ^4^Beijing Advanced Innovation Center for Big Data-Based Precision Medicine, School of Medicine, Beihang University, Beijing, China; ^5^Department of Radiology, Shanghai Pulmonary Hospital, Tongji University School of Medicine, Shanghai, China

**Keywords:** computed tomography, radiomics, non-small-cell lung cancer, tyrosine kinase inhibitor resistance, anaplastic lymphoma kinase

## Abstract

**Objectives:** To identify a computed tomography (CT)-based radiomic signature for predicting progression-free survival (PFS) in stage IV anaplastic lymphoma kinase (*ALK*)-positive non-small-cell lung cancer (NSCLC) patients treated with tyrosine kinase inhibitor (TKI) crizotinib.

**Materials and Methods:** This retrospective proof-of-concept study included a cohort of 63 stage IV *ALK*-positive NSCLC patients who had received TKI crizotinib therapy for model construction and validation. Another independent cohort including 105 stage IV *EGFR*-positive NSCLC patients was also used for external validation in *EGFR*-TKI treatment. We initially extracted 481 quantitative three-dimensional features derived from manually segmented tumor volumes of interest. Pearson's correlation analysis along with the least absolute shrinkage and selection operator (LASSO) penalized Cox proportional hazards regression was successively performed to select critical radiomic features. A CT-based radiomic signature for PFS prediction was obtained using multivariate Cox regression. The performance evaluation of the radiomic signature was conducted using the concordance index (C-index), time-dependent receiver operating characteristic (ROC) analysis, and Kaplan–Meier survival analysis.

**Results:** A radiomic signature containing three features showed significant prognostic performance for *ALK*-positive NSCLC patients in both the training cohort (C-index, 0.744; time-dependent AUC, 0.895) and the validation cohort (C-index, 0.717; time-dependent AUC, 0.824). The radiomic signature could significantly risk-stratify *ALK*-positive NSCLC patients (hazard ratio, 2.181; *P* < 0.001) and outperformed other prognostic factors. However, no significant association with PFS was captured for the radiomic signature in the *EGFR*-positive NSCLC cohort (log-rank tests, *P* = 0.41).

**Conclusions:** The CT-based radiomic features can capture valuable information regarding the tumor phenotype. The proposed radiomic signature was found to be an effective prognostic factor in stage IV *ALK* mutated nonsynchronous nodules in NSCLC patients treated with a TKI.

## Introduction

Non-small-cell lung cancer (NSCLC) accounts for 85% in lung cancer (1), making it the leading cause of cancer-related mortality across the globe (2). An approximate estimate of 5% NSCLC patients are found to have rearrangements in the anaplastic lymphoma kinase (*ALK*) gene. Although the incidence of *ALK* seems relatively low among NSCLC patients, a number of nearly 40,000 *ALK*-positive cases occur annually worldwide (3), and about half of them come from Asia.

Crizotinib, which is regarded as the first-line treatment for stage IV NSCLC patients with *ALK* gene rearrangements according to the clinical practice guidelines given by the American Society of Clinical Oncology (ASCO) (4), has a proven therapeutical efficacy. And those who respond to crizotinib may show rapid improvement in symptoms, including cough, dyspnea, and pain (5, 6).

In spite of the potency of crizotinib, however, some patients still suffered from the disease progression, and the median survival time just ranged from 7 months to 1 year (4, 7, 8). Some of the reported mechanisms underlying the acquired resistance to crizotinib in *ALK* translocated cancers involve secondary mutations, *ALK* amplification, KIT amplification, and autophosphorylation of epidermal growth factor receptor (*EGFR*) (9–13). Therefore, at the genetic level, resistance to tyrosine kinase inhibitor (TKI) is hard to detect (14).

In patients with *ALK* rearrangements, when disease progresses after crizotinib therapy, other *ALK* inhibitor (alectinib or brigatinib or ceritinib) or local therapy (e.g., stereotactic ablative radiotherapy or surgery) is recommended by the National Comprehensive Cancer Network (NCCN) guidelines (15). Thus, if the progression on crizotinib can be predicted, other *ALK* inhibitor or local therapy can be considered in the initial systemic therapy strategy, which will help the individualized treatment decision-making. Therefore, finding a prognostic marker to predict the development of TKI resistance of *ALK*-positive patients will be of great significance.

Using automatic feature extraction algorithms, radiomics is capable of converting embedded information in medical images into mineable data (16, 17), which has been widely applied in the prediction of preoperative distant metastasis, histologic subtype classification, and so on (18–20). Prognosis based on radiomics is gaining popularity as associations between radiomic features and the underlying genomic patterns emerge in various cancers (21–24). On one hand, a latest study showed that a subset of radiomic features were able to consistently capture texture information about the underlying tissue histology, but some of them were incapable to be observed at the purely human level (25, 26). On the other hand, computed tomography (CT) imaging modality can quantify the tissue density and is widely used in radiology researches.

Gene mutations in adenocarcinoma include *EGFR* mutation, kirsten rat sarcoma viral oncogene (KRAS) mutation, *ALK* mutation or c-ros oncogene 1 receptor kinase (ROS1) rearrangement, etc. *EGFR* mutations occupy the majority of gene mutations in adenocarcinoma. Previous studies ever used radiomics to predict progression-free survival (PFS) in stage IV *EGFR*-mutant NSCLC patients with *EGFR*-TKI therapy (24). However, as another mutation independent of *EGFR*, no studies have yet been conducted to investigate the potential of radiomics in the prediction of *ALK* inhibitor-related PFS in *ALK* rearranged patients with stage IV NSCLC.

Consequently, this proof-of-concept study aimed to launch and verify a radiomic signature to predict PFS in stage IV *ALK*-positive NSCLC patients treated with TKI crizotinib, and further investigate the association of this radiomic signature with PFS in *EGFR*-mutant NSCLC patients with *EGFR*-TKI therapy.

## Materials and Methods

### Patients

From January 2012 to January 2017, a total of 63 stage IV *ALK*-positive NSCLC patients (33 males and 30 females) and 105 stage IV *EGFR*-positive NSCLC patients (42 males and 63 females) were enrolled. This study was examined and approved by the review committee as a retrospective research. Confidential information for each patient has been hidden. The inclusion criteria for *ALK*-positive patients were as follows: (1) histopathologically confirmed stage IV NSCLC according to the TNM classification system of the American Joint Committee on Cancer (AJCC); (2) available test results for *ALK* mutation status; and (3) underwent contrast-enhanced CT scans 2 weeks before the crizotinib therapy. Patients were ruled out for any of the following reasons: (1) underwent other antitumor therapies such as resection for local advanced or metastatic disease; and (2) death for other reasons during follow-up visits. A rapid prescreening for *ALK* mutation was done by immunohistochemistry (IHC). Positive findings were confirmed subsequently by fluorescence *in situ* hybridization (FISH) analysis (27, 28). The inclusion criteria for *EGFR*-positive patients were as follows: (1) histopathologically confirmed stage IV NSCLC according to the TNM classification system of the AJCC; (2) available data for the *EGFR* mutation status; and (3) underwent contrast-enhanced CT scans 2 weeks before the *EGFR*-TKI treatment. Patients were ruled out for any of the following reasons: (1) underwent other antitumor therapies such as resection for locally advanced or metastatic disease; and (2) death for other reasons during follow-up visits. Note that patients with synchronous nodules were not included in the study.

PFS was counted from the start of treatment with TKI to the confirmation of disease progression or death. Basic clinical characteristics including sex, age, and smoking status were recorded. The clinical characteristics and follow-up information for patients are illustrated in Table 1.

**Table 1:** Baseline demographic and clinicopathologic characteristics of patients in the training and validation cohorts.

**Characteristic**	**Training cohort (*n* = 32)**	**Validation cohort (*n* = 31)**	***P*-value**
Sex, No. (%)			0.895
Male	16 (50)	17 (55)	
Female	16 (50)	14 (45)	
Smoking status, No. (%)			0.954
Smoker	5 (16)	6 (19)	
Nonsmoker	27 (84)	25 (81)	
Age (years), No. (%)			0.714
≤49	13 (41)	15 (48)	
>49	19 (59)	16 (52)	
Progression, No. (%)			0.853
Yes	21 (66)	22 (71)	
No	11 (34)	9 (29)	

*Demographic and clinicopathologic characteristics of patients with ALK-positive non-small-cell lung cancer in the training and validation cohorts. Statistical comparisons between the training and validation cohorts were computed using the χ^2^ test. A P < 0.05 indicates a significant difference between the two cohorts. ALK, anaplastic lymphoma kinase*.

### CT Image Acquisition and Tumor Segmentation

CT scans were examined after a 60-s delay following an intravenous injection of 100 ml Iopromide (Ultravist-300; Bayer Schering Pharma, Berlin Germany) at a rate of 3 ml/s for enhancement. The scanning parameters were 120 kV, 160 mAs, 0.6 s rotation time, and a matrix of 512×512. Portal venous phase CT images were reconstructed using a slice thickness of 1 mm.

A radiologist engaged in the chest CT interpretation for more than 7 years segmented the primary tumors of all the patients slice by slice. Three-dimensional (3D) manual segmentation was performed using the ITK-SNAP software (www.itksnap.org, version 3.6.0).

### Statistical Analysis

**Figure 1:**
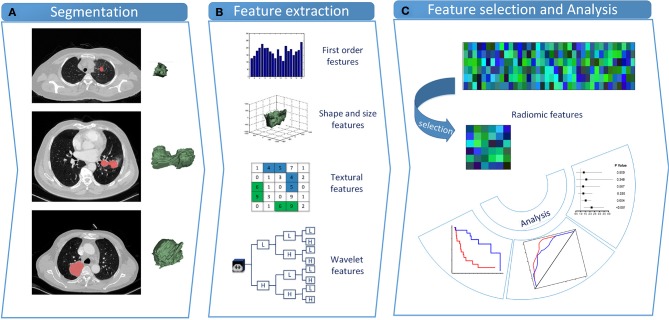
Study design. **(A)** Tumor segmentation. Shown are typical CT images of lung cancer patients with tumor contours and three-dimensional visualizations. **(B)** Radiomic feature extraction. Four types of radiomic features were extracted from VOIs. **(C)** Feature selection and statistical analysis. LASSO penalized Cox proportional hazards regression was adopted to select critical features. CT, computed tomography; VOI, volume of interest; LASSO, least absolute shrinkage and selection operator.

The designed workflow of the study is shown in Figure 1. All statistical tests were conducted using the R software (www.r-project.org, version 3.5.1). A summary of R packages adopted in the study can be found in Supplementary Appendix 1. To ensure that there were enough cases left in the validation cohort for model evaluation, we initially separated the dataset into the training cohort and the validation cohort at a 1:1 ratio. Besides, we further performed 10 random cohort allocations and conducted feature selections in the training cohorts to test the stability of the radiomic features to be selected. *EGFR*-positive NSCLC patients were used as another independent validation cohort. Features were initially normalized. Radiomic feature selection and radiomic signature construction were carried out based on the training cohort, whereas the validation cohorts were only for performance verification.

### Radiomic Feature Extraction

Within each tumor volume of interest (VOI), 481 radiomic features across four feature types were extracted from 3D CT images. These characteristics embodied first-order, shape, texture, and wavelet features. Feature extraction procedures were conducted within the VOIs with in-house algorithms implemented in Matlab R2018b (The MathWorks, Natick, MA, USA).

First-order features characterized the distribution of intensity values, according to commonly used and basic metrics such as energy, entropy, kurtosis, etc. 3D shape descriptors were included to describe the shape of the tumor VOI, which was independent of the gray-level intensity distribution. Volume, surface area, sphericity, compactness, and other features were embodied. Gray-level co-occurrence matrix (GLCM) (29) described the second-order joint probability function of an image region, and gray-level run-length matrix (GLRLM) (30, 31) calculated texture features that represented voxel intensities of spatial distribution or patterns. Wavelet features effectively decoupled information via decomposing the original image. Details about feature extraction algorithms and parameters are given in Supplementary Appendix 2.

### Feature Discovery and Radiomic Signature Building

Based on Harrell's guideline, the number of features must be reduced to <1/10 of the events in the multivariate Cox regression (32). Therefore, Pearson correlation analysis and the least absolute shrinkage and selection operator (LASSO) penalized Cox proportional hazards regression were successively performed to reduce the number of radiomic features and select critical ones. In the training cohort, the Pearson correlation coefficient matrix of all radiomic features was calculated. An absolute value of correlation coefficient >0.8 indicated a strong correlation between the two features, one of which was removed then. The LASSO penalized Cox proportional hazards regression was adopted to select critical features with nonzero weights. According to minimizing the partial likelihood deviance, an optimal value of λ was adopted via the 10-fold cross-validation in the training cohort. Finally, the radiomic signature was built by fitting the selected critical features using multivariate Cox regression.

### Validation of Radiomic Signature

Associations between the radiomic signature and PFS were evaluated in both *ALK*-mutant cohorts. Based on the median radiomic signature value generated in the training cohort, patients were classified into high and low radiomic signature value groups, which were expected to represent rapid and slow progression treated with crizotinib, respectively. When not all the subjects continue to be followed up in the study, Kaplan–Meier survival curve is usually one of the best choices to be used to measure the fraction of subjects living for a certain amount of time after treatment. At the same time, the log-rank test is always used to obtain the statistical significance level in survival difference between two groups. In this study, the potential association between the radiomic signature and PFS was assessed by Kaplan–Meier survival curves as well as log-rank tests (G-rho rank test, rho = 1). The discriminative ability of the radiomic signature was assessed by time-dependent receiver operating characteristic (ROC) analysis and the Harrell's concordance index (C-index). Due to the relatively short survival time range of stage IV NSCLC patients in this study, time-dependent ROCs were plotted in both cohorts at 6 months. Furthermore, to identify whether our model had the prognostic value in other treatments, we constructed an EGFR-mutant cohort to further validate the radiomic signature by Kaplan–Meier survival curves along with log-rank tests.

### Performance Assessment and Comparison of Prognostic Factors

To further verify the prognostic performance of the radiomic signature and test the prognostic ability of clinical characteristics, we established a clinical model. Aiming to investigate whether the combination of clinical characteristics and radiomic signature could further improve the model performance, we also established a combined model via multivariate Cox proportional hazards regression. C-index statistics were calculated to assess the model performance. Herein, the outputs of the two models could be deemed two prognostic factors for stage IV *ALK*-positive NSCLC patients treated with TKI crizotinib.

Moreover, to find the optimal prognostic factor, the association of each prognostic factor with PFS, including each clinical characteristic, the radiomic signature, the clinical model (constructed using significant clinical characteristics), and the combined model, was analyzed and compared with respect to the hazard ratio (HR) and corresponding *P*-values in the validation cohort, respectively. *P-*values are computed using the likelihood ratio test to measure whether the HR is different from 1. The forest plots of HR were conducted.

## Results

### Clinical Characteristics and PFS

In the *ALK*-mutant cohorts, a total of 63 patients who met the inclusion and exclusion requirements were enrolled, all of whom were stage IV *ALK*-mutant NSCLC treated with TKI crizotinib. The last follow-up was on April 30, 2016. Throughout the follow-up period, 43 patients experienced progression (21 patients in the training cohort, 22 patients in the validation cohort), and 20 patients were lost to follow-up (11 patients in the training cohort, 9 patients in the validation cohort). The PFS was balanced between the training cohort (median PFS, 248 days) and the validation cohort (median PFS, 265 days). Furthermore, there were no differences between the two cohorts with respect to baseline clinical characteristics including age, sex, and smoking status by chi-square tests (Table 1).

In the *EGFR*-mutant cohort, we included 105 patients who met the enrollment requirements, all of whom were stage IV *EGFR*-mutant NSCLC treated with TKI treatment. Throughout the follow-up period, the median PFS was 238 days.

### Radiomic Signature Building

**Figure 2:**
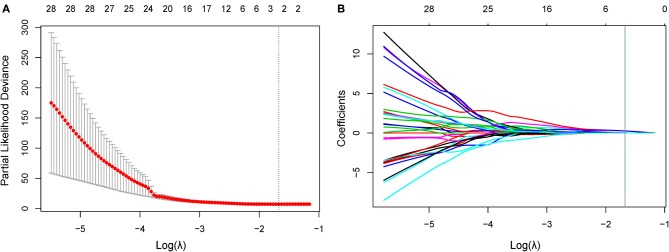
Feature selection using the LASSO Cox model. **(A)** In the LASSO Cox model, the minimum standard is adopted to obtain the value of the super parameter λ by 10-fold cross-validation. The λ value was confirmed as 0.1876. **(B)** Shown here is a coefficient sectional view plotted against the log (λ) magnitude. Based on 10-fold cross-validation, the optimal λ corresponding to three nonzero coefficients were obtained where the vertical line was drawn. LASSO, least absolute shrinkage and selection operator.

In the training cohort, 34 features were retained after Pearson's correlation analysis. Then, cluster shade and inverse variance calculated from GLCM were selected by the LASSO Cox regression. The value of parameter λ was 0.1876 (Figure 2). The three selected radiomic features stayed stable in the training cohorts from 10 random cohort allocations (Supplementary Appendix 3). Finally, the formula for the radiomic signature generated by multivariate Cox regression was shown below:

$$\begin{array}{l} \text{Radiomic Signature }=\;0.312\times \text{first order minimum}\times \text{cluster }\\ \text{ shade}+0.602\times \text{short run high gray }\\ \text{ level emphasis }\left(\text{SRHGLE}\right) \end{array}$$

### Validation of Radiomic Signature

**Figure 3:**
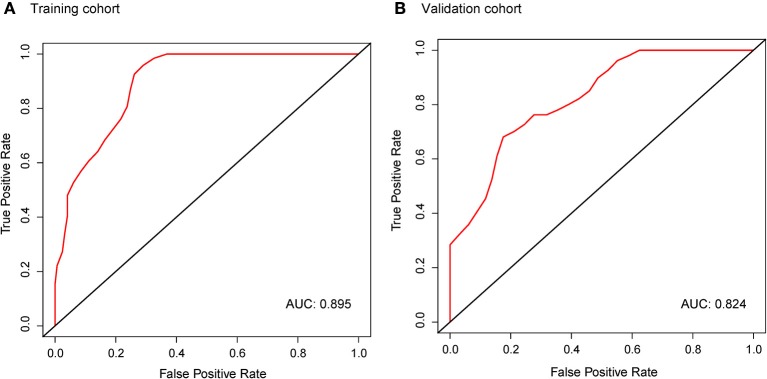
Time-dependent ROC curves of the radiomic signature in the training cohort **(A)** and validation cohort **(B)** at 6 months. AUCs were used to assess the prognostic accuracy in both cohorts. ROC, receiver operating characteristics; AUC, area under the curve.

In the *ALK*-mutant patients, the radiomic signature was relevant to PFS in the training cohort [HR, 2.718; 95% confidence interval (CI): 1.599–4.621; *P* < 0.01] and the validation cohort (HR, 2.181; 95% CI: 1.370–3.471; *P* < 0.01). The C-index yielded from the radiomic signature was 0.744 (95% CI: 0.678–0.809) in the training cohort and 0.717 (95% CI: 0.614–0.821) in the validation cohort. Moreover, in the 10 random validation cohorts, the mean of the C-index was 0.709 (range: 0.648–0.759). Time-dependent ROC curves achieved a training area under the curve (AUC) of 0.895 at 6 months and a validation AUC of 0.824 (Figure 3).

**Figure 4:**
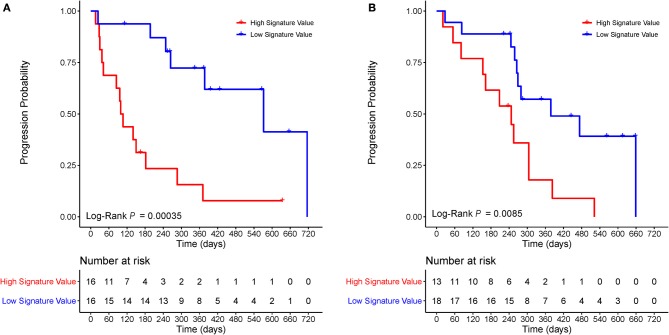
Kaplan–Meier survival curves of *ALK*-positive NSCLC patients. The *P*-values were calculated using log-rank tests. Kaplan–Meier survival analysis for patients stratified by median radiomic signature in the training **(A)** and validation **(B)** cohorts showed a significant association between the radiomic signature and PFS. *ALK*, anaplastic lymphoma kinase; NSCLC, non-small-cell lung cancer; PFS, progression-free survival.

Based on the cutoff value of the median radiomic signature in the training cohort, we allocated patients into high and low signature value groups. Thereinto, the high signature value group (median survival: training cohort, 107 days; validation cohort, 208 days) had a shorter median PFS than the low signature value group (median survival: training cohort, 377 days; validation cohort, 279 days). In both cohorts, the PFS differed significantly between the high and low signature value groups based on Kaplan–Meier survival curves (Figure 4) with log-rank *P*-values of 0.00035 and 0.0085, respectively.

**Figure 5:**
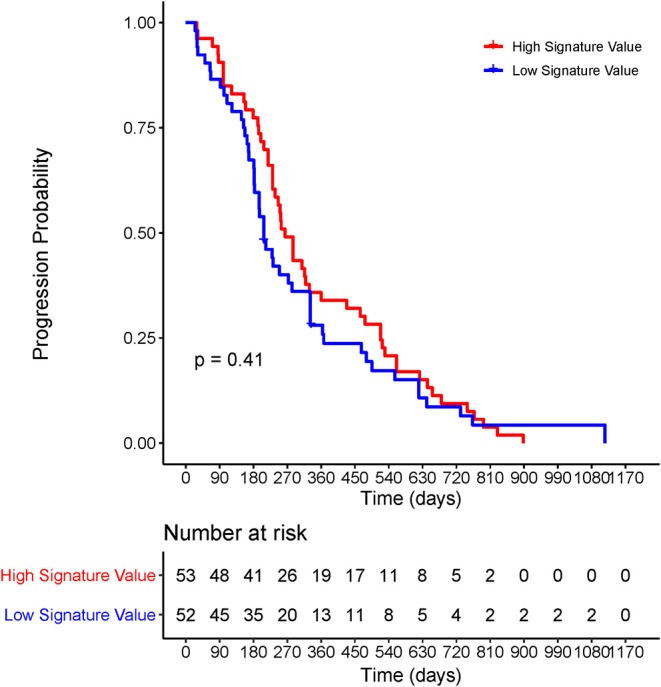
Kaplan–Meier survival curve of the radiomic signature for *EGFR*-positive NSCLC patients. The *P*-values were calculated using log-rank test. *EGFR*, epidermal growth factor receptor; NSCLC, non-small-cell lung cancer; PFS, progression-free survival.

In the *EGFR*-mutant cohort, however, the radiomic signature failed to significantly risk-stratify NSCLC patients by Kaplan–Meier survival curves with a log-rank *P*-value of 0.41 (Figure 5).

### Performance Analysis and Comparison of Prognostic Factors

Since the smoking status and age were found to be significantly correlated with PFS in the training cohort (*P* = 0.045 and *P* = 0.028, respectively), a clinical model was built by the two characteristics. The C-index yielded from the clinical model was 0.638 (95% CI: 0.498–0.778) in the training cohort and 0.525 (95% CI: 0.416–0.634) in the validation cohort. A trial was made to feed the smoking status, age, and the radiomic signature into a multivariate Cox regression, the result of which showed that adding the clinical characteristics did not result in an extra benefit compared with the radiomic signature alone, with a C-index of 0.789 (95% CI: 0.694–0.885) in the training cohort and 0.655 (95% CI: 0.614–0.821) in the validation cohort. We also conducted the feature selection on both radiomic features and clinical variables; only the radiomic features were reserved in the model.

**Figure 6:**
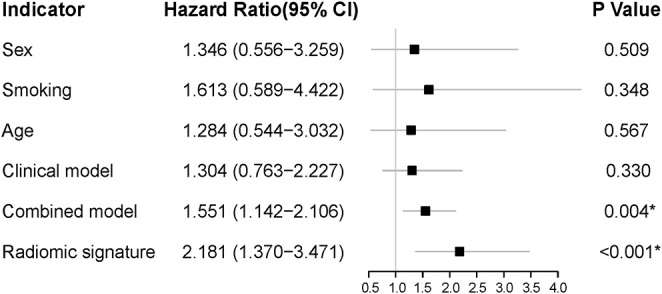
The forest plots of HR for each clinical characteristic, each selected radiomic feature, the radiomic signature, the clinical model, and the combined model in the validation cohort. The *P*-value was computed using the likelihood ratio test, and a *P < 0.05 was considered statistically significant. HR, hazard ratio.

The forest plots showed that the radiomic signature had the significant correlation with PFS in the validation cohort (*P* < 0.001). The combined model, which was built by the clinical characteristics and radiomic signature, also showed a significant correlation with PFS, but it had a much higher *P-*value than the radiomic signature alone (Figure 6). Thereinto, the radiomic signature also demonstrated superior prognostic efficacy to the clinical characteristics (sex, age, and smoking status), the clinical model (constructed using smoking status and age), and the combined model.

## Discussion

A CT-based radiomic signature was identified as an independent prognostic factor to predict PFS in stage IV *ALK*-positive NSCLC patients treated with TKI crizotinib. The radiomic signature managed to risk-stratify the patients into groups of rapid progression and slow progression. Our results showed no significant correlation between PFS and the radiomic signature in *EGFR*-mutant NSCLC patients, indicating the specific stratification ability of the radiomic signature in predicting *ALK*-TKI resistance. In addition, adding the clinical characteristics did not result in an extra benefit compared with the radiomic signature alone. The clinical model built by clinical characteristics failed to predict the prognosis as well, which demonstrated that the three clinical characteristics (sex, age, and smoking status) could not predict the prognosis in *ALK*-positive NSCLC treated with TKI crizotinib.

To our knowledge, the correlations between CT-based radiomic features and PFS in *ALK*-positive NSCLC patients treated with crizotinib have not been evaluated. The patients with a shorter PFS would particularly benefit if we could predict their response to crizotinib therapy and offer prompt guidance for alternative treatment strategies. For stage IV patients with rapidly progressed tumors, a platinum-based regimen is helpful (33). There is evidence showing that early palliative care together with standard care improved the overall mood, quality of life, and even survival in NSCLC patients, although these patients received less aggressive treatments compared to patients receiving standard care alone (34). Several ongoing studies are evaluating treatment options for *ALK*-positive patients. Crizotinib therapy can be continued for *ALK*-positive patients showing disease progression as long as they do not have multiple systemic symptomatic lesions (35). Ceritinib was suggested as a follow-up treatment by NCCN Panel for *ALK*-positive NSCLC patients who progressed after crizotinib therapy. Patients with *ALK*-positive metastatic NSCLC who have progressed or cannot tolerate crizotinib can also be treated with an oral TKI alectinib, which is approved by the FDA (36). Our novel radiomic signature showed potential in identifying patients with high and low risk of disease progression, and hence could guide the course of treatment. For patients who are prone to crizotinib resistance, it can be compared between the curative effects of targeted agents after chemotherapy and the first-line *ALK* inhibitor. Besides, although a new generation of TKIs are being promoted, the size of clinical data available for research is still very small. More researches on other TKI resistance may be inspired by the outcomes of this study in the future.

Radiomics, a novel and noninvasive method, can capture information from the entire tumor quantitatively and remove the obstacles hindering the prediction of PFS in patients with the same stage disease. In the process of building a radiomic signature, 481 features were reduced to two components using Pearson's correlation analysis and the LASSO Cox regression. Pearson's correlation analysis could greatly reduce the redundancy of the data by eliminating highly correlated features. The LASSO method is ideal for analyzing cases when the sample size is smaller than the feature dimension, and it generally gives accurate results and avoids overfitting (37, 38). Thus, the critical features obtained were combined as a radiomic signature, revealing sufficient discrimination in both cohorts. The selected radiomic features including cluster shade and SRHGLE are both texture features. Cluster shade measures the skewness and the uniformity of the GLCM. The higher the cluster shade, the greater the asymmetry about the mean. SRHGLE represents the measurement of the joint distribution of higher gray-level shorter run lengths of the GLRLM. They may reflect tumor heterogeneity in two ways, and a higher value of cluster shade or SRHGLE may indicate higher tumor heterogeneity. The interpretations between the two texture features and tumor heterogeneity need further investigation. Besides, in terms of the multivariate Cox regression formula, more rapid resistance to crizotinib is more likely to be caused by tumors with high heterogeneity.

This study still has some limitations, given the fact that the study is retrospective. Since the sample size was small and only Asian data sets were included, this proof-of-concept study needs further verification on large-scale cohorts. We excluded patients with synchronous nodules, which are commonly seen among NSCLC patients; further study on synchronous nodules should be performed in the future. In the *EGFR* cohort, not only one kind of crizotinib was used, the influence of which should be further studied. In addition, further prospective trials are in desperate need to be carried out to assure the predictive efficacy of the CT-based radiomic signature in TKI resistance.

In conclusion, this study discussed the correlation of a radiomic signature with PFS and its predictive potential in stage IV *ALK*-positive NSCLC patients treated with crizotinib. Besides, it can predict resistance to the *ALK* inhibitor, thereby enabling the effective prognostic prediction and stratification, which can improve the individualized treatment with *ALK* inhibitors.

## Data Availability Statement

The datasets for this article are not publicly available because of patient information privacy. Requests to access the datasets should be directed to Prof. Jie Tian, jie.tian@ia.ac.cn.

## Ethics Statement

The studies involving human participants were reviewed and approved by the review committee of Shanghai Pulmonary Hospital. Written informed consent for participation was not required for this study in accordance with the national legislation and the institutional requirements.

## Author Contributions

JT, JS, and DD conceived and launched this study. HL, SW, and RZ designed the medical and statistical analyses. JS, ZH, and DD collected cases and implemented the control of image quality and clinical diagnosis. HL, MF, and YZ analyzed the data and carried out statistical experiments. HL, SW, MF, and JS provided result interpretation. HL, SW, and RZ wrote the first draft of this manuscript. DD, ZH, JT, and JS revised and edited the final version. All the authors reviewed and approved the manuscript.

### Conflict of Interest

The authors declare that the research was conducted in the absence of any commercial or financial relationships that could be construed as a potential conflict of interest.

## Abbreviations

CT: computed tomography
PFS: progression-free survival
ALK: anaplastic lymphoma kinase
NSCLC: non-small-cell lung cancer
TKI: tyrosine kinase inhibitor
LASSO: least absolute shrinkage and selection operator
C-index: concordance index
ASCO: American Society of Clinical Oncology
EGFR: epidermal growth factor receptor
NCCN: National Comprehensive Cancer Network
ICC: interclass correlation coefficient
VOI: volume of interest
GLCM: gray-level co-occurrence matrix
GLRLM: gray-level run-length matrix
ROC: receiver operating characteristic
HR: hazard ratio
CI: confidence interval
AUC: area under the curve.
