# Supplementary material for: CT-Based Radiomic Signature as a Prognostic Factor in Stage IV ALK-Positive Non-small-cell Lung Cancer Treated With TKI Crizotinib: A Proof-of-Concept Study
Source: Front Oncol. 2020 Feb 18;10:57. doi: 10.3389/fonc.2020.00057 (PMC7040202; doi:10.3389/fonc.2020.00057)
Supplement: Supplementary file 1 [file Data_Sheet_1.PDF]

Supplementary Appendix

1 R packages

R software (version 3.5.1) was used in this study. Least absolute shrinkage and selection operator (LASSO) Cox regression was performed using the “glmnet” package. Multivariate Cox regression models were constructed with the “survival” package. C-index calculation was performed using the “survcomp” package. The “survival ROC” package was used for the time-dependent ROC curve analysis. The Kaplan-Meier survival analysis was performed using the “survminer” package. The forest plots were plotted using “forestplot” package.

2 Radiomic features

Radiomic features used in this paper consisted of four types: first-order features, shape and size features, texture features, and wavelet features. First-order features characterize the distribution of intensity values, according to commonly used and basic metrics including energy, entropy, kurtosis, maximum, mean, mean absolute deviation, median, minimum, range, root mean square (RMS), skewness, standard deviation, uniformity, and variance. Three-dimensional shape and size descriptors were included to describe the shape of the volume of interest, which were independent from the gray level intensity distribution in the region of interest (ROI). This type of features consists of compactness, maximum three-dimensional diameter, spherical disproportion, sphericity, surface area, surface to volume ratio, and volume. Some of them describe the size of the ROI and others provided information on how spherical, rounded or the shape of the region. For texture features, gray-level co-occurrence matrix (GLCM) based features are made up of autocorrelation, cluster prominence, cluster shade, cluster tendency, contrast, correlation, difference entropy, dissimilarity, energy, entropy, homogeneity, informational measure of correlation, inverse difference normalized, inverse variance, maximum probability, sum average, sum entropy, sum variance, and variance. Gray-level run length matrix (GLRLM) based features are listed as below: short run emphasis, long run emphasis, gray level non-uniformiy, run length non-uniformity, run percentage, low gray level run emphasis, high gray level run emphasis, short run low gray level emphasis, short run high gray level emphasis, long run low gray level emphasis, and long run high gray level emphasis. Texture features based on GLCM matrix describe the second-order joint probability function of an image region and GLRLM matrix represent patterns or the spatial distribution of voxel intensities. Wavelet features effectively decouple information by decomposing the original image. We calculated the above first-order and texture features on each decomposition.

LASSO (Tibshirani, 1996) method is a compression estimate. It constructs a refined model by constructing a penalty function so that it compresses some coefficients by setting some coefficients to zero. Thus, preserving the advantage of subset shrinkage, which is a biased estimate of dealing with multicollinearity data. The basic idea of LASSO is to minimize the sum of squares of residuals under the constraint that the sum of the absolute values of the regression coefficients is less than a constant, and thus produce some regression coefficients that are strictly equal to zero to obtain an interpretable model.

The method reduces correlations among discovered variables to prevent over fitting, which is represented by the penalty parameter (λ). The larger the value of λ, the fewer the number of selected

predictors. We used the LASSO Cox regression model after Mann-Whitney U test to select the best potential prognostic features from all the features in the training cohort. The elastic-net mixing parameter (alpha) was set to 1 and the number of lambda (nlambda) was set to 100. A multi-feature-based radiomic signature was constructed to risk stratify stage IV anaplastic lymphoma kinase (ALK)-positive non-small-cell lung cancer (NSCLC) patients treated with tyrosine kinase inhibitor (TKI) crizotinib in the training cohort.

The selected radiomic features are calculated as follows [1, 2].

The detailed names of the features we selected are X2_fos_minimum, X1_GLCM_cluster_shade, and X0_GLRLM_SRHGLE, where "Xn" means different wavelet filters, "fos" represents "first-order statistics".

Minimum: The minimum intensity value of the three-dimensional image matrix.

A GLCM is defined as $P(i,j;\delta,\alpha)$, a matrix with size $N_g \times N_g$ describing the second-order joint probability function of an image, where the (i,j)th element represents the number of times the combination of intensity levels i and j occur in two pixels in the image, that are separated by a distance of δ pixels in direction α, and N_g is the number of discrete gray level intensities.

Let:

$P(i,j)$ be the co-occurrence matrix for an arbitrary δ and α,

N_g be the number of discrete intensity levels in the image,

μ be the mean of $P(i,j)$,

$p_x(i) = \sum_{j=1}^{N_g} P(i,j)$ be the marginal row probabilities,

$p_y(i) = \sum_{i=1}^{N_g} P(i,j)$ be the marginal column probabilities,

μ_x be the mean of p_x,

μ_y be the mean of p_y,

Cluster Shade:

$$cluster\ shade = \sum_{i=1}^{N_g} \sum_{j=1}^{N_g} \left[i + j - \mu_x(i) - \mu_y(j)\right]^3 P(i,j)$$

A GLRLM is defined as the length in number of pixels, of consecutive pixels that have the same gray level value. In a gray level run length matrix $P(i,j;\theta)$, the (i,j)th element describes the number of times j a gray level i appears consecutively in the direction specified by θ, and N_g is the number of discrete gray level intensities.

Let:

$P(i,j;\theta)$ be the (i,j)th entry in the given run-length matrix p for a direction θ,

N_g the number of discrete intensity values in the image,

N_r the number of different run lengths,

N_p the number of voxels in the image.

Short Run High Gray Level Emphasis (SRHGLE)

$$SRHGLE = \frac{\sum_{i=1}^{N_g}\sum_{j=1}^{N_r}[\frac{p(i,j|\theta)i^2}{j^2}]}{\sum_{i=1}^{N_g}\sum_{j=1}^{N_r}p(i,j|\theta)}$$

3 Random cohort allocation tests

Considering the small dataset size in this study, we performed additional ten random cohort allocations to ensure that the stability of models was not affected by the allocation of training and validation cohorts. The experimental results were as follows.

No.	The selected features	The number of the same or relevant features	C-index<br>in validation cohort
1	"X4_fos_maximum"*<br>"X1_GLCM_cluster_shade"*<br>"X5_GLCM_inverse_variance"*	3	0.747<br>(95%CI: 0.662-0.832)
2	"Sph_dis"<br>"X2_fos_minimum"*<br>"X1_GLCM_cluster_shade"*<br>"X5_GLCM_inverse_variance"*	3	0.686<br>(95%CI: 0.577-0.796)
3	"X1_fos_minimum"<br>"X0_GLCM_autocorrelation"<br>"X1_GLCM_cluster_shade"*<br>"X3_GLRLM_HGLRE"*	2	0.682<br>(95%CI: 0.573-0.792)
4	"X6_fos_maximum" *<br>"X1_GLCM_cluster_shade"*<br>"X0_GLRLM_SRHGLE"*	3	0.722<br>(95%CI: 0.6200-0.824)
5	"X4_fos_maximum"<br>"X0_GLCM_inverse_variance"*	1	0.658<br>(95%CI: 0.547-0.769)

6	"Max3D"*<br>"X2_fos_range"*<br>"X5_GLCM_inverse_variance"*<br>"X8_GLCM_sum_average"<br>"X5_GLRLM_SRE"	3	0.713<br>(95%CI: 0.605-0.821)
7	"X2_fos_minimum"*<br>"X1_GLCM_cluster_shade"*<br>"X0_GLRLM_SRHGLE"*<br>"X1_GLRLM_mean"	3	0.757<br>(95%CI: 0.678-0.837)
8	"X2_fos_range" *<br>"X1_GLCM_cluster_shade"*<br>"X0_GLRLM_SRHGLE"*	3	0.759<br>(95%CI: 0.673-0.845)
9	"X0_GLCM_cluster_shade"*<br>"X2_GLCM_inverse_variance"*<br>"X5_GLCM_sum_average"<br>"X5_GLRLM_SRE"	2	0.648<br>(95%CI: 0.506-0.789)
10	"X8_fos_mean"*<br>"X0_GLCM_cluster_shade"*<br>"X5_GLCM_inverse_variance"<br>"X8_GLCM_sum_average"<br>"X5_GLRLM_SRE"	2	0.719<br>(95%CI: 0.598-0.840)

NOTE. * represents the same or relevant (Pearson's correlation coefficients, $|r| > 0.3$) features compared with those selected in the manuscript (X2_fos_minimum, X1_GLCM_cluster_shade, and X0_GLRLM_SRHGLE). CI, Concordance Index.

References

[1] Aerts H J W L, Velazquez E R, Leijenaar R T H, et al. Decoding tumour phenotype by noninvasive imaging using a quantitative radiomics approach[J]. Nature communications, 2014, 5: 4006.

[2] Van Griethuysen J J M, Fedorov A, Parmar C, et al. Computational radiomics system to decode the radiographic phenotype[J]. Cancer research, 2017, 77(21): e104-e107.
